# Tolerance to induced astigmatism of patients with trifocal or extended depth of focus intraocular lens implantation

**DOI:** 10.3389/fmed.2024.1462205

**Published:** 2024-08-23

**Authors:** Jiancheng Mu, Tianxu Xiong, Feng Xu, Wanyue Guo, Chuhuan Sun, Hao Chen, Wei Fan

**Affiliations:** Department of Ophthalmology, West China Hospital of Sichuan University, Chengdu, Sichuan, China

**Keywords:** astigmatism, cataracts, presbyopia-correcting, intraocular lens, tolerance, visual quality

## Abstract

**Background:**

Residual astigmatism is common after cataract surgery involving implantation of an intraocular lens, yet the tolerance of presbyopia-correcting intraocular lens to astigmatism of different magnitudes and axes is poorly understood. Here we compared visual acuity and quality in the presence of induced astigmatism after implantation of a trifocal or extended-depth-of-focus (EDOF) intraocular lens, the two widely used presbyopia-correcting intraocular lenses.

**Methods:**

At least 3 months after implantation of a TFNT00 or ZXR00 intraocular lens, patients were analyzed by slit-lamp examination, non-contact tonometry, subjective refraction, iTrace aberrometry, and corneal topography. After correction of residual astigmatism, astigmatism of different magnitudes on different axes was induced using cylindrical lenses, and overall visual acuity was measured, while objective visual quality was measured using the Optical Quality Analysis System II. Subjects were also asked about subjective visual quality using the Visual Function-14 questionnaire.

**Results:**

Comparison of 18 individuals who received a trifocal lens and 19 who received an EDOF lens showed that objective visual quality was better in the EDOF group regardless of the magnitude or axis of the induced astigmatism. In both groups, astigmatism of at least −1.00 DC influenced distant vision more severely when the axis was 45° than 0° or 90°, meanwhile astigmatism of at least −1.50 DC influenced near and intermediate vision more severely when the axis was 45° than 0° or 90°.

**Conclusion:**

Trifocal or EDOF intraocular lenses are less tolerant of oblique astigmatism than astigmatism with or against the rule. EDOF lenses may provide better objective visual quality than trifocal lenses in the presence of astigmatism, regardless of its magnitude or axis.

## Introduction

Cataracts, which affect up to 17% of the global population at any one time, are the most frequent cause of reversible blindness (1). Prognosis for individuals with cataracts has improved tremendously through medical advances, with phacoemulsification and implantation of an intraocular lens restoring good vision quality to many. Increasingly attractive are intraocular lenses that correct presbyopia, such as trifocal and extended-depth-of-focus (EDOF) lenses, because they can obviate the need for glasses after cataract surgery (2). While these lenses can provide excellent near, intermediate and distant vision, they are associated with higher risk of adverse visual phenomena, such as glare and halos, than monofocal lenses are (3, 4). In addition, residual astigmatism after cataract surgery is quite common, exceeding 1.00 DC in up to 56% of patients in one study (5), and it is unclear to what extent presbyopia-correcting lenses are tolerant of residual astigmatism. Previous studies have compared the tolerance to induced astigmatism between small-aperture, mono-or multi-focal intraocular lenses implanted in pseudophakic eyes (6–8), but we are unaware of tolerance comparisons between trifocal and EDOF intraocular lenses implanted into pseudophakic eyes. Establishing the astigmatism tolerance of trifocal and EDOF lenses is important because residual astigmatism as low as 0.75 DC can reduce satisfaction with vision after cataract surgery (9).

Here we examined such tolerance in individuals after implantation of trifocal or EDOF lenses by inducing astigmatism of different magnitudes along different axes and then measuring overall visual acuity and visual quality.

## Methods

### Study participants

The protocol for this study was approved by the Ethics Review Board of West China Hospital, Sichuan University (approval 1,312, 2021), and the study was registered on December 15, 2021 in the Chinese Clinical Trial Register (ChiCTR2100054362). Participants were prospectively recruited from among all adults scheduled for cataract surgery at the Department of Ophthalmology of West China Hospital between May 2020 and October 2022 who (a) had nuclear or cortical cataracts without posterior polar cataracts or concomitant intraocular disease, (b) had preoperative intraocular pressure < 21 mmHg, and (c) elected to undergo implantation with a PanOptix TFNT00 trifocal intraocular lens (Alcon Laboratories, Fort Worth, TX, United States) or the Tecnis Symfony ZXR00 EDOF intraocular lens (Johnson & Johnson Vision, Santa Ana, CA, United States) during cataract surgery. After being thoroughly informed about the functions of different IOLs, patients choose the type of intraocular lens based on their individual conditions.

We excluded patients who had (a) pre-or postoperative abnormality of the cornea, macula, or optic nerve; (b) postoperative development of secondary cataracts or significant intraocular lens displacement; (c) pre-or postoperative ocular inflammation; (d) history of ocular surgery; or (e) postoperative intraocular pressure > 21 mmHg. We also excluded patients who failed to complete follow-up.

### Preoperative examinations

Prior to cataract surgery, all patients were examined for uncorrected distance visual acuity (UDVA), best corrected distant visual acuity (BCDVA), uncorrected and corrected near visual acuity, refraction, intraocular pressure based on non-contact tonometry, biometrics based on partial coherence interferometry (IOL Master 700, Carl Zeiss Meditec, Jena, Germany), corneal tomography (CASIA 2, Tomey, Nagoya, Japan) and topography (Topographic Modeling System, Tomey, Nagoya, Japan). The macula and retina were examined using optical coherence tomography (Heidelberg Engineering, Heidelberg, Germany) and scanning laser ophthalmoscopy (Optos, Marlborough, MA, United States), while corneal aberration was evaluated using an iTrace visual function analyzer (Tracey Technologies, Houston, TX, United States).

### Cataract surgery and intraocular lens implantation

The surgeries on all patients were performed by the same experienced clinician using the Stellaris system (Bausch & Lomb, Rochester, NY, United States). After topical anesthesia and pupillary dilation, cataract surgery was performed with a clear corneal self-sealing incision 2.0 mm long, continuous curvilinear capsulorhexis with a diameter of 5.0–5.5 mm, hydro-dissection and-delineation, phacoemulsification, irrigation and aspiration of the residual lens cortex, and insertion of either TFNT00 or ZXR00 lens.

After surgery, all patients were instructed to take eye drops containing 0.3% tobramycin and 0.1% dexamethasone (Alcon-Couvreur, Puurs, Belgium) four times a day during week 1, three times a day during week 2, twice a day during week 3, and once a day during week 4. All patients were also asked to take eye drops containing 0.1% sodium diclofenac (Sinqi Pharmaceutical, Shenyang, Liaoning, China) four times a day for 4–6 weeks depending on the degree of postoperative inflammatory response.

### Follow-up and assessment of astigmatism tolerance

All patients were followed up according to routine procedures in our department. For the present report, a single set of measurements from a follow-up visit conducted at least 3 months (actually ranged from 6 months to 1 year) after surgery was analyzed. Uncorrected near, intermediate and distant visual acuity were measured, and refractive examination, non-contact tonometry, slit-lamp examination and corneal topography were performed. The modulation transfer function and wavefront aberrations were measured at a pupil diameter of 4 mm using the iTrace aberrometer. Subjects were asked to rate their subjective visual experience and satisfaction on the Visual Function-14 questionnaire (10).

Residual refractive errors were corrected, then BCDVA, best intermediate visual acuity (BIVA) and best near visual acuity (BNVA) were measured. Subjects were asked to wear cylindrical lenses that induced astigmatism of −1.00, −1.50 or-2.00 DC along the axes of 0° (“with the rule”), 45° (“oblique”) or 90° (“against the rule”). Under each of the nine situations, visual acuity was measured at near (40 cm), intermediate (60 cm) and far (5 m) distances using international standard logarithmic near-, intermediate-, and far-vision visual acuity charts. Results were converted to the LogMAR visual acuity scale for statistical analysis. Acuity was tested immediately after inducing astigmatism to avoid neural adaptation (11).

Objective visual quality was measured in terms of the modulation transfer function cutoff frequency (MTF cutoff), Strehl ratio (SR), and objective scatter index (OSI) using a dual-channel Optical Quality Analysis System II (Visiometrics, Terrassa, Spain). Contribution of residual refractive errors to these three measurements was removed by using additional lenses or the system’s built-in low-order aberration correction. Visual quality was measured under the nine situations of induced astigmatism as described above.

Before measuring objective visual quality, we ensured that pupil diameter exceeded 4 mm, and we asked subjects to blink several times to ensure that the ocular surface was uniformly covered by tears.

### Statistical analysis

Data were analyzed using SPSS 26.0 (Chicago, IL, United States) and GraphPad Prism 8.3.0 (GraphPad Software, San Diego, CA, United States). Continuous data were reported as mean ± standard deviation if normally distributed, or as median (interquartile range) if skewed. Differences in continuous, normally distributed variables were assessed for significance using the independent-samples *t*-test in the case of pairwise comparisons, or using ANOVA in the case of comparisons involving at least three groups. Differences in continuous, skewed variables were assessed for significance using the Mann–Whitney *U* test in the case of pairwise comparisons, or using the Kruskal–Wallis test followed by Dunn’s method in the case of comparisons involving at least three groups. Categorical data were reported as frequency (percentage). Pairwise differences in categorical variables were assessed for significance using the chi-squared test if the expected frequency in either group was greater than 5, or using Fisher’s exact test otherwise. A sample size of 16 subjects provided adequate power to detect this difference at a significance level of 0.05 using a two-sided paired *t*-test. Differences were considered statistically significant if associated with *p* < 0.05.

## Results

The final analysis included 37 eyes from 37 participants, comprising 18 eyes in the trifocal group and 19 in the EDOF group. The two groups did not differ significantly in any of the preoperative characteristics examined, except that the trifocal group showed a significantly longer axial length (Table 1). This is consistent with such individuals’ greater requirement for good near vision.

**Table 1:** Preoperative characteristics of individuals undergoing cataract surgery involving implantation of a trifocal or EDOF intraocular lens.

Characteristic	Trifocal group	EDOF group	p*
No. eyes/patients	18/18	19/19	
Age, yr	62.5 ± 6.4	62.3 ± 7.3	0.775
Sex			0.495
Male	7 (39)	5 (26)	
Female	11 (61)	14 (74)	
Axial length, mm	25.3 ± 1.91	23.94 ± 1.73	0.046
Anterior chamber depth, mm	3.24 ± 0.43	3.16 ± 0.33	0.165
Corneal refractive power, D	43.67 ± 1.18	44.05 ± 1.65	0.136
Corneal astigmatism, D	0.7 ± 0.4	0.46 ± 0.28	0.113

Values are *n*, *n* (%) or mean ± SD, unless otherwise noted. EDOF, extended depth of focus; IQR, interquartile range (quartile 1, quartile 3); SD, standard deviation. *Based on Fisher’s exact test for sex or the independent-samples *t-*test for all other variables.

Postoperative data for all study participants were collected at follow-up visits that occurred at least 3 months after cataract surgery.

### Comparison of the trifocal and EDOF groups in the absence of induced astigmatism

In the absence of induced astigmatism, the trifocal and EDOF groups did not differ significantly in postoperative refraction or in any of the corneal parameters examined, including curvature, astigmatism or spherical aberration (Table 2). Similarly, the two groups did not differ significantly on any of the items on the Visual Function-14 questionnaire (Table 3), although the EDOF group tended to report higher incidence of starbursts, while the trifocal group tended to report higher incidence of glare and halos. Nevertheless, the two groups reported similarly high satisfaction with visual function provided by their implanted lens.

**Table 2:** Postoperative examination results of individuals at least 3 months after cataract surgery involving implantation of a trifocal or EDOF intraocular lens.

Characteristic	Trifocal group (*n* = 18)	EDOF group (*n* = 19)	p*
Spherical diopter, D	0.03 ± 0.32	−0.22 ± 0.42	0.118
Cylindrical diopter, D	−0.28 ± 0.45	−0.32 ± 0.30	0.663
Equivalent spherical diopter, D	−0.13 ± 0.36	−0.36 ± 0.35	0.057
Corneal refraction, D	43.67 ± 1.17	44.09 ± 1.68	0.101
Corneal astigmatism, D	0.69 ± 0.41	0.53 ± 0.29	0.128
κ angle, mm	0.285 ± 0.178	0.207 ± 0.124	0.105
α angle, mm	0.318 ± 0.152	0.405 ± 0.146	0.105
Corneal spherical aberration, D	0.224 ± 0.057	0.226 ± 0.089	0.245
Total ocular higher-order aberration, μm	0.135 ± 0.097	0.157 ± 0.181	0.845
Corneal higher-order aberrations, μm	0.102 ± 0.126	0.085 ± 0.060	0.461
Intraocular pressure, mmHg	13.2 ± 1.9	13.3 ± 1.9	0.964

Values are mean ± SD. EDOF, extended depth of focus. *Based on the independent-samples *t-*test in the case of the three corneal parameters of curvature, astigmatism and spherical aberration; or based on the non-parametric Mann–Whitney *U* test in the case of other parameters.

**Table 3:** Responses on the Visual Function-14 questionnaire and self-report of other visual function measures at least 3 months after cataract surgery involving implantation of a trifocal or EDOF intraocular lens.

Score or measure	Trifocal group (*n* = 18)	EDOF group (*n* = 19)	p*
Visual Function-14 questionnaire scores**			
Total	95.95 ± 9.50	94.94 ± 12.97	0.167
Distant tasks	96.94 ± 8.24	96.36 ± 10.62	0.707
Intermediate tasks	98.15 ± 6.61	99.56 ± 3.31	0.500
Near tasks	96.76 ± 8.48	99.12 ± 4.64	0.297
Fine tasks	88.57 ± 14.02	77.63 ± 20.79	0.063
Incidence of adverse visual effects			
Starbursts	7 (38.9)	8 (42.1)	1.000
Glare	5 (27.8)	2 (10.5)	0.232
Halos	8 (44.4)	6 (31.6)	0.508
Satisfaction with postoperative visual function***	90.56 ± 7.25	91.58 ± 8.34	0.649
Proportion of individuals reporting satisfaction ≥90 points	14 (77.8)	14 (73.7)	1.000
Likelihood of recommending the same intraocular lens to family or friends***	87.78 ± 23.15	91.58 ± 17.4	0.279
Proportion of individuals reporting ≥90% likelihood of recommending the same lens	14 (77.8)	15 (78.9)	1.000

Values are *n* (%) or mean ± SD, unless otherwise noted. EDOF, extended depth of focus. *Based on Fisher’s exact test in the case of the incidence of starbursts, glares and halos; or based on the Mann–Whitney *U* tests in all other cases. **Original scores, which could vary from a minimum of 0 to a maximum of 4, were converted to a 100-point scale. Higher score indicates better visual perception. ***Original scores on a 10-point scale were converted to a 100-point scale.

**Figure 1:**
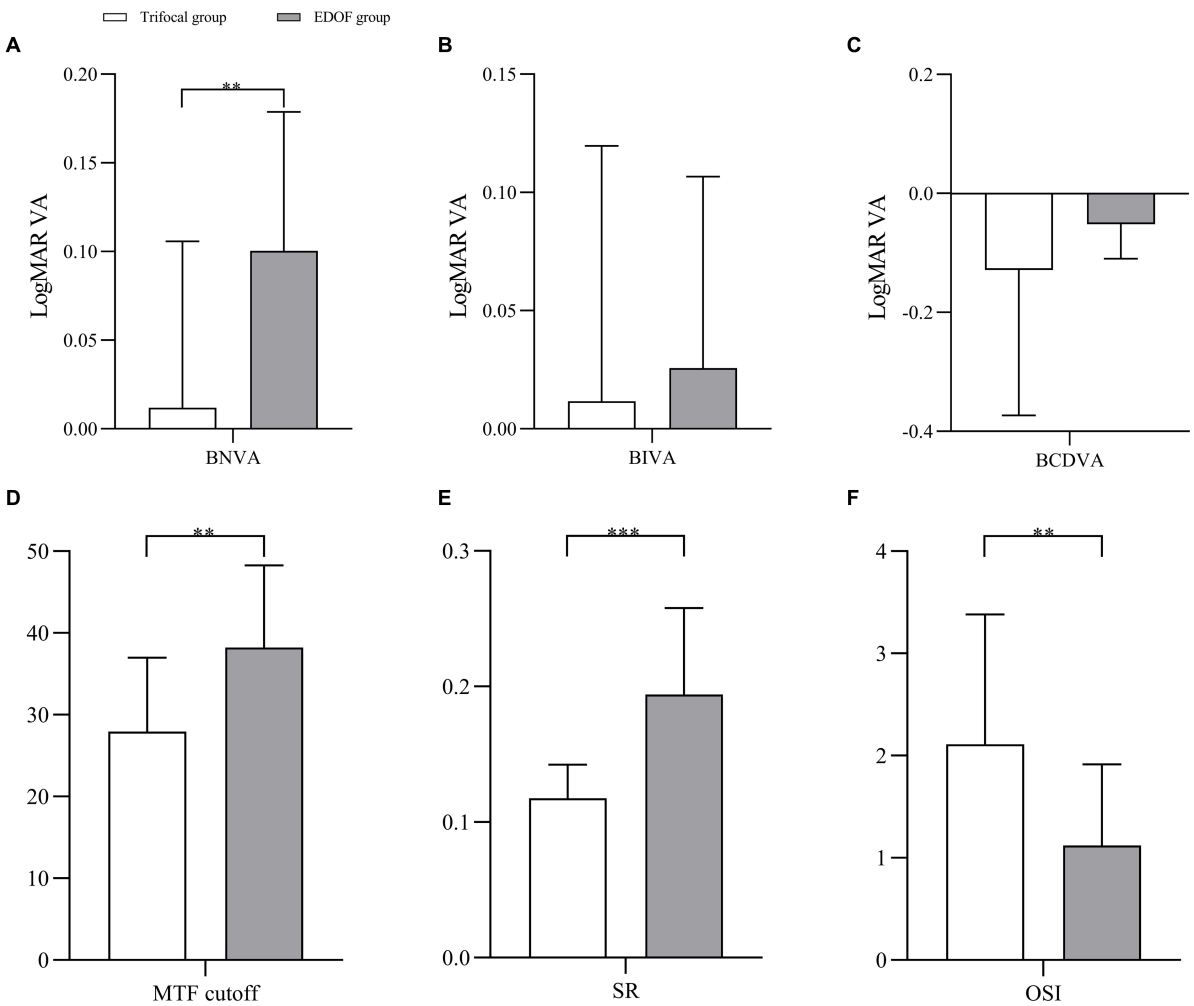
Pairwise comparison of **(A)** near vision, **(B)** intermediate vision, **(C)** distant vision, **(D)** MTF cutoff, **(E)** SR, and **(F)** OSI between individuals who received trifocal or EDOF intraocular lenses in absence of induced astigmatism. BNVA, best near visual acuity; BIVA, best intermediate visual acuity; BCDVA, best corrected distant visual acuity; EDOF, extended depth of focus; LogMAR VA, visual acuity in terms of the logarithm of the minimum angle of resolution; MTF, modulation transfer function; OSI, objective scatter index; SR, Strehl ratio. *p < 0.05, **p < 0.01, ***p < 0.001, ****p < 0.0001.

In the absence of induced astigmatism, the trifocal group showed significantly better BNVA than the EDOF group but the two groups did not differ significantly in UDVA, BCDVA or BIVA (Figures 1A–C). The EDOF group showed significantly better modulation transfer function cutoff, Strehl ratio and objective scatter index (Figures 1D–F). In fact, mean values of all three parameters were within the normal range in the EDOF group but not in the trifocal group. Nevertheless, the two groups showed similar uncorrected visual acuity and reported similar satisfaction with their visual function.

### Comparison of the trifocal and EDOF groups in the presence of induced astigmatism

**Figure 2:**
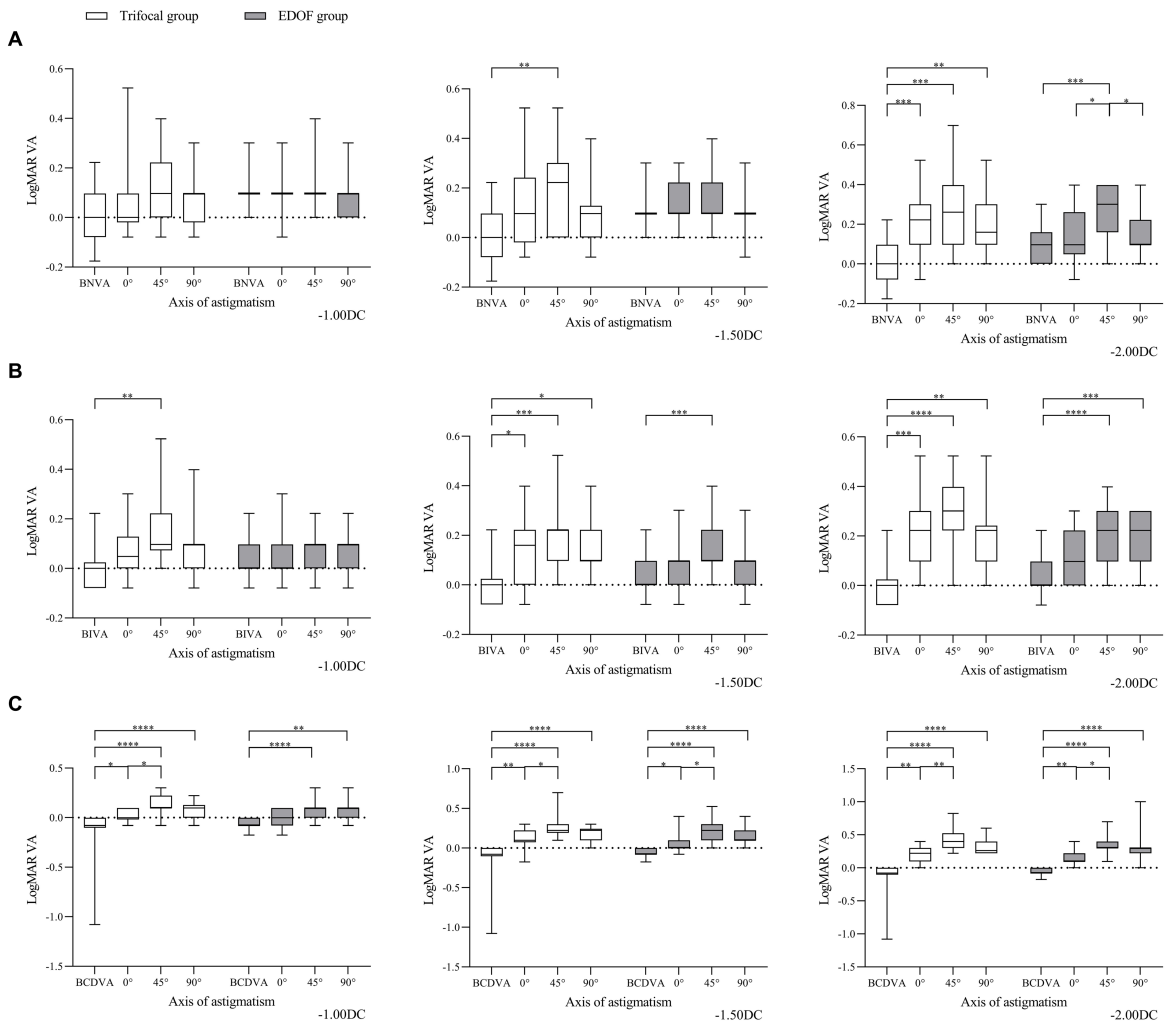
Comparison of best corrected acuity of **(A)** near vision, **(B)** intermediate vision and **(C)** distant vision under the specified magnitudes of induced astigmatism at different axes between individuals who received trifocal or EDOF intraocular lenses. Measurements were made after astigmatism of −1.00, −1.50 or −2.00 DC was induced at the indicated axis values at least 3 months after implantation. BCDVA, best corrected distant visual acuity; BIVA, best intermediate visual acuity; BNVA, best near visual acuity; EDOF, extended depth of focus; LogMAR VA, Logarithm of the Minimum Angle of Resolution visual acuity. *p < 0.05, **p < 0.01, ***p < 0.001, ****p < 0.0001.

Induced astigmatism of −1.00 DC did not significantly affect the acuity of near vision in either group, regardless of whether the axis was 0, 45 or 90° (Figure 2). It slightly affected intermediate vision, primarily in the trifocal group when the axis was 45°. In contrast, it significantly diminished distant vision in both groups when the axis was 45 or 90°; only distant vision in the trifocal group was significantly reduced when the axis was 0°.

Induced astigmatism of −1.50 DC did not significantly affect near vision in the EDOF group, regardless of the axis, whereas it did significantly affect near vision in the trifocal group at an axis of 45°. Similarly, it significantly reduced intermediate vision in the EDOF group only at an axis of 45° but in the trifocal group at all three axis values. It significantly reduced distant vision in both groups, regardless of the axis, with the most severe reduction occurring at an axis of 45°.

Induced astigmatism of-2.00 DC significantly reduced near vision in the trifocal group regardless of the axis, but in the EDOF group only at an axis of 45°. Similarly, it significantly reduced intermediate vision in the trifocal group regardless of the axis, but in the EDOF group only at axes of 45 or 90°. It significantly reduced distant vision in both groups, regardless of the axis, with an axis of 45° associated with more severe reduction than 0°.

**Figure 3:**
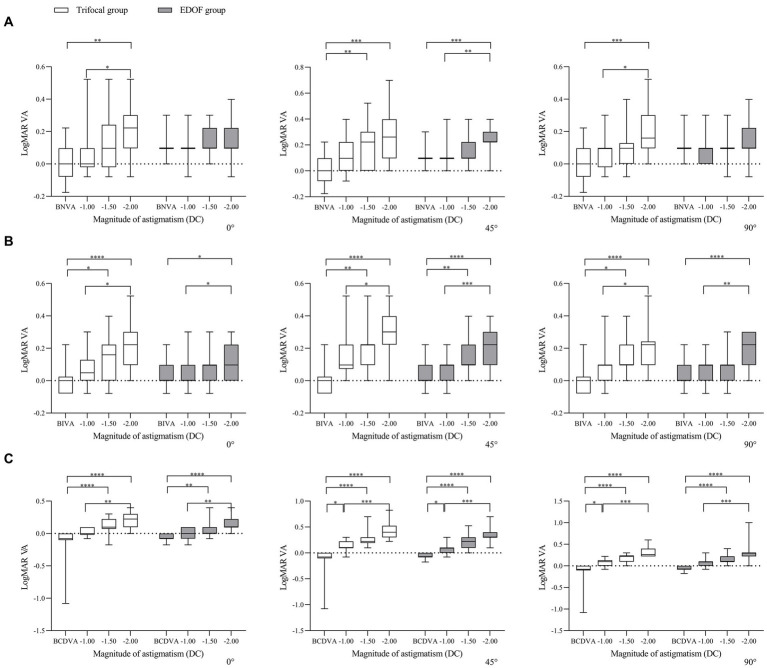
Comparison of best corrected acuity of **(A)** near vision, **(B)** intermediate vision and **(C)** distant vision under induced astigmatism of different magnitudes at the specified axes between individuals who received trifocal or EDOF intraocular lenses. Measurements were made after astigmatism of −1.00, −1.50 or −2.00 DC was induced at the indicated axis values at least 3 months after implantation. BCDVA, best corrected distant visual acuity; BIVA, best intermediate visual acuity; BNVA, best near visual acuity; EDOF, extended depth of focus; LogMAR VA, Logarithm of the Minimum Angle of Resolution visual acuity. *p < 0.05, **p < 0.01, ***p < 0.001, ****p < 0.0001.

In other words, when the axis of induced astigmatism was 0°, the trifocal group experienced significant loss of near vision only at −2.00 DC, whereas the EDOF group did not experience significant loss even at that magnitude (Figure 3). The trifocal group experienced significant loss of intermediate vision from −1.50 DC, compared to −2.00 DC in the EDOF group. Both groups experienced significant loss of distant vision from −1.50 DC. When the axis of induced astigmatism was 45°, both groups experienced significant loss of near and intermediate vision from −1.50 DC, and they experienced significant loss of distant vision already from −1.00 DC. When the axis of induced astigmatism was 90°, similar to when the axis was 0°, the trifocal group experienced significant loss of near vision only at −2.00 DC, whereas the EDOF group did not experience significant loss even at that magnitude. The trifocal group experienced significant loss of intermediate vision from −1.50 DC, compared to −2.00 DC in the EDOF group. In contrast to when the axis was 0°, the trifocal group experienced significant loss of distant vision from −1.00 DC, compared to −1.50 DC in the EDOF group.

**Figure 4:**
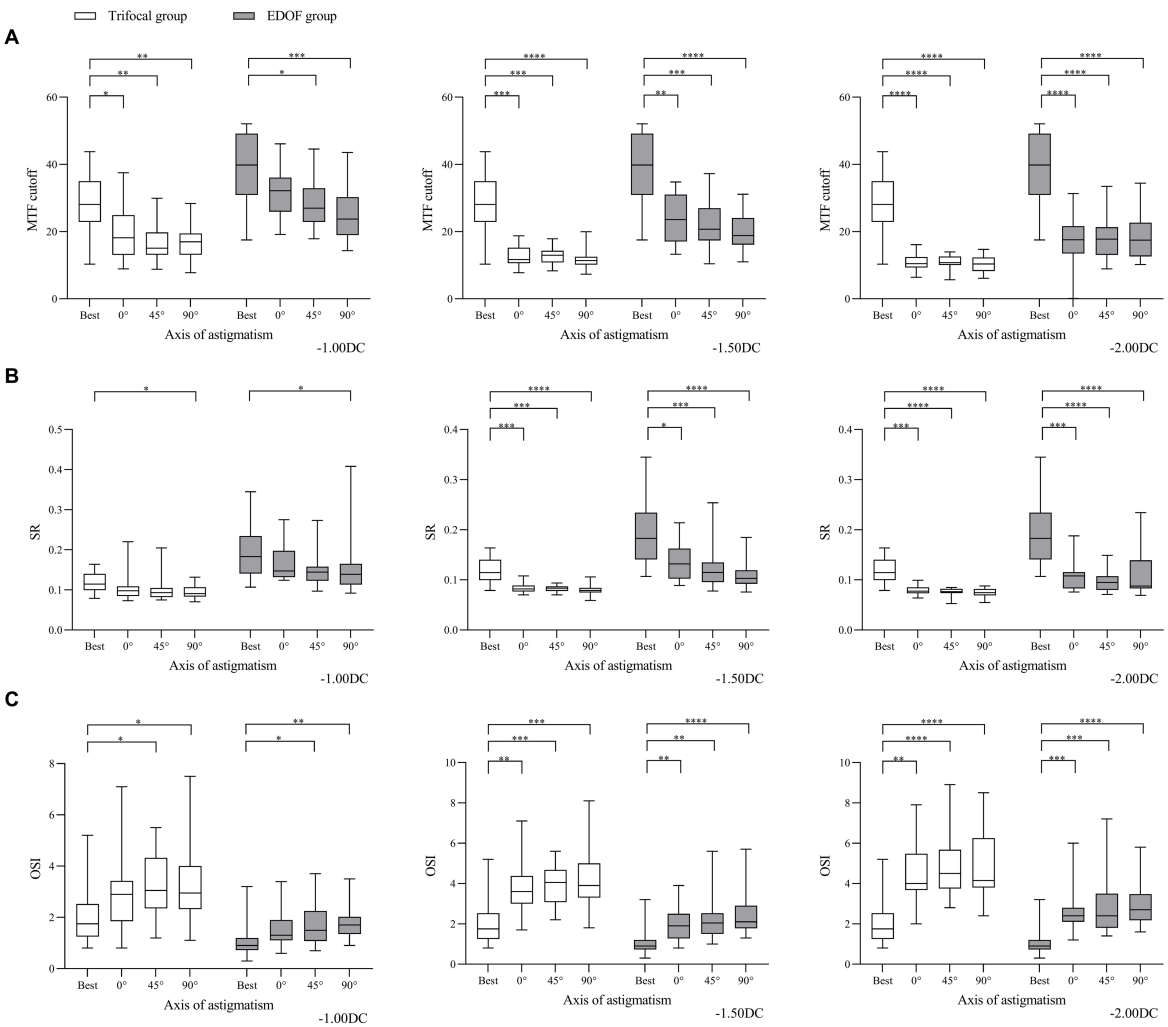
Comparison of objective visual quality under the specified magnitudes of induced astigmatism at different axes between individuals who received trifocal or EDOF intraocular lenses in terms of **(A)** MTF cutoff, **(B)** OSI and **(C)** SR. Measurements were made after astigmatism of −1.00, −1.50 or −2.00 DC was induced at the indicated axis values at least 3 months after implantation. EDOF, extended depth of focus; MTF, modulation transfer function; OSI, objective scatter index; SR, Strehl ratio. *p < 0.05, **p < 0.01, ***p < 0.001, ****p < 0.0001.

Consistent with these findings on visual acuity, we found that objective visual quality, as measured in terms of modulation transfer function, Strehl ratio and objective scatter index, was better for the EDOF group than the trifocal group in the presence of induced astigmatism. Astigmatism of −1.00 DC significantly reduced the modulation transfer function cutoff in both groups at axis values of 45° or 90°, while it also reduced the cutoff at an axis of 0° in the trifocal group (Figure 4). In contrast, the same magnitude of astigmatism significantly reduced the Strehl ratio in both groups, but only when the axis was 90°; it significantly reduced the objective scatter index in both groups only when the axis was 45 or 90°. Regardless of axis, induced astigmatism of −1.50 or −2.00 DC significantly reduced all three visual quality parameters in both groups.

**Figure 5:**
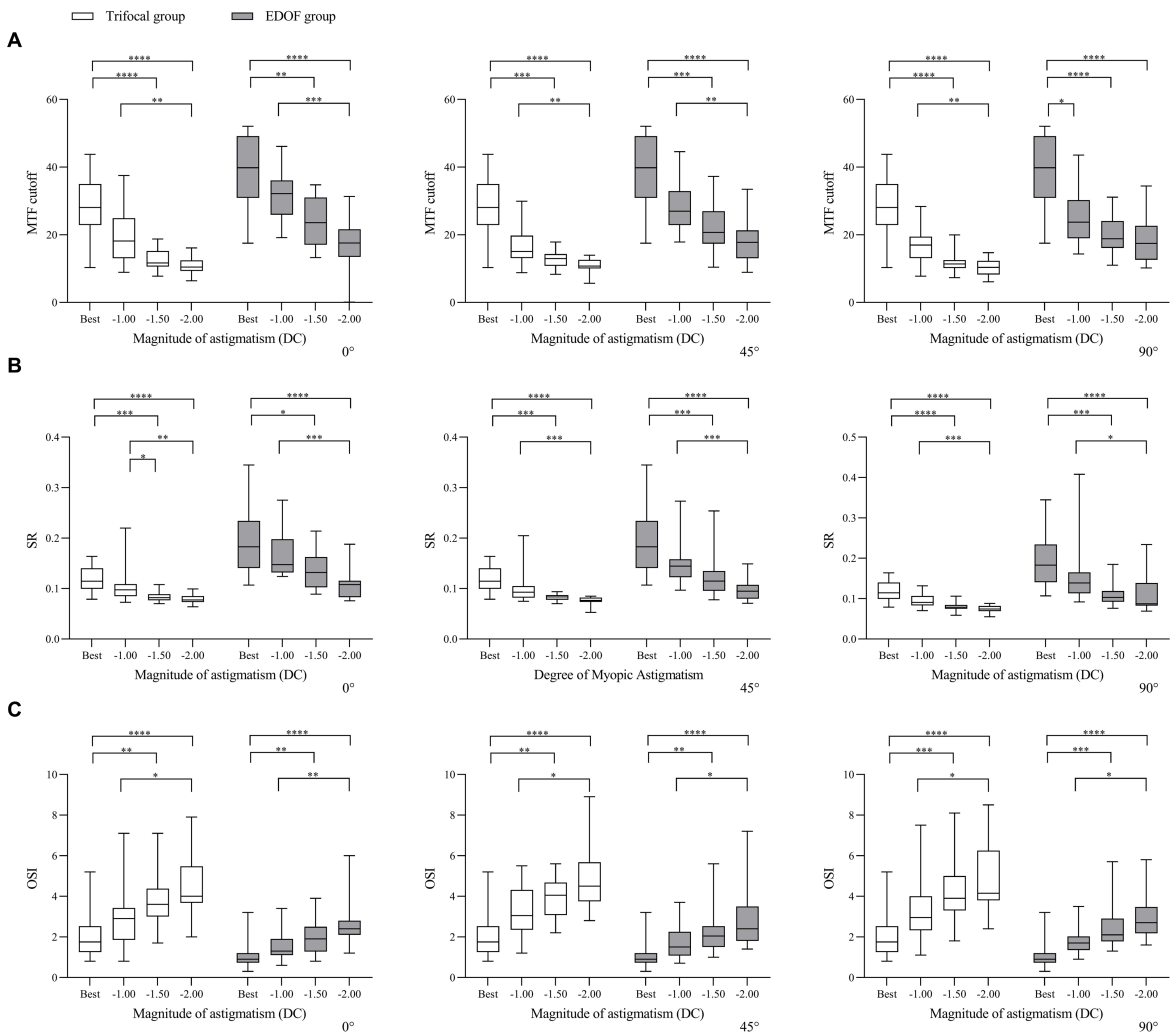
Comparison of objective visual quality under induced astigmatism of different magnitudes at the specified axes between individuals who received trifocal or EDOF intraocular lenses in terms of **(A)** MTF cutoff, **(B)** OSI and **(C)** SR. Measurements were made after astigmatism of −1.00, −1.50 or −2.00 DC was induced at the indicated axis values at least 3 months after implantation. EDOF, extended depth of focus; MTF, modulation transfer function; OSI, objective scatter index; SR, Strehl ratio. *p < 0.05, **p < 0.01, ***p < 0.001, ****p < 0.0001.

In other words, regardless of the axis of induced astigmatism, the three visual quality parameters in both groups declined significantly from −1.50 or −2.00 DC (Figure 5). When the axis was 90°, the only parameter to decline significantly in the EDOF group was modulation transfer function cutoff from −1.00 DC.

**Figure 6:**
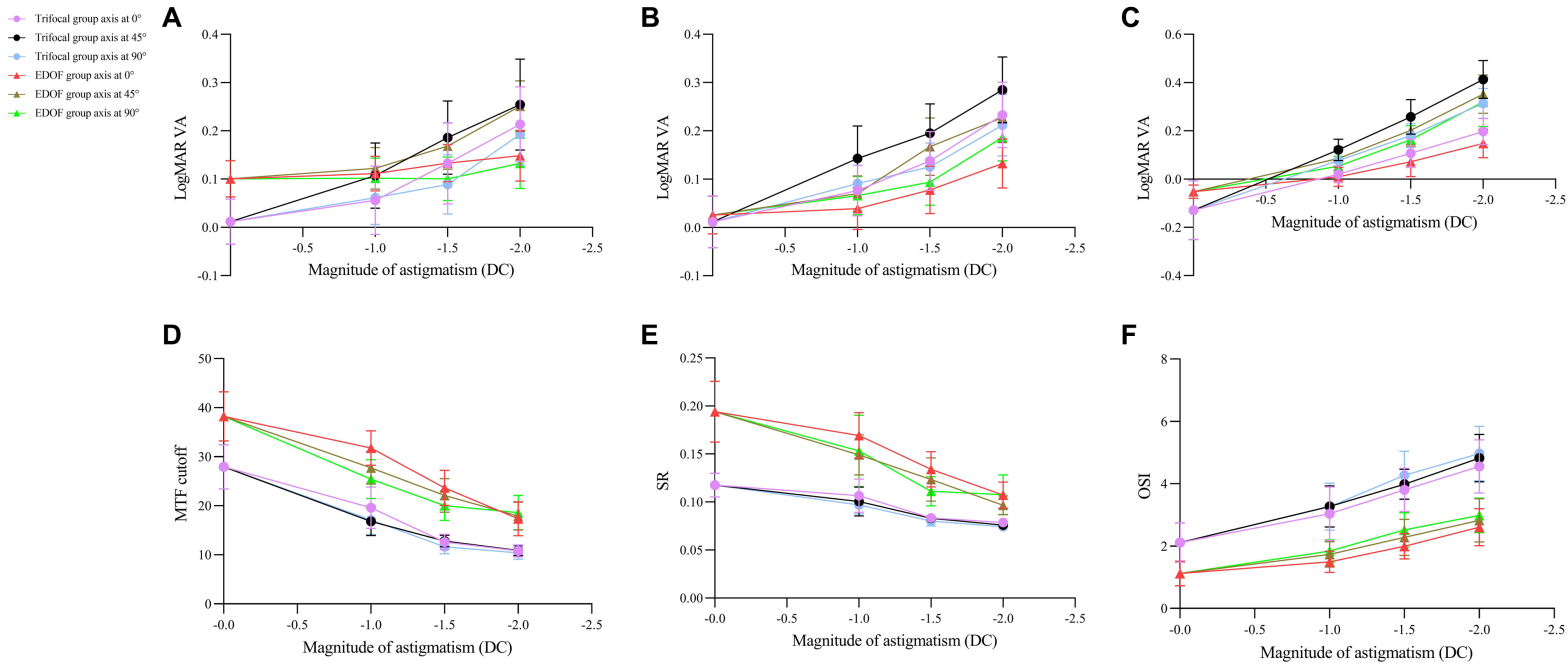
Dependence of visual acuity and quality on magnitude of induced astigmatism in individuals who received trifocal or EDOF intraocular lenses. Measurements were made after astigmatism of −1.00, −1.50 or −2.00 DC was induced at axis values of 0, 45, or 90° at least 3 months after implantation. The following parameters were assessed: **(A)** near visual acuity, **(B)** intermediate visual acuity, **(C)** distant visual acuity, **(D)** MTF cutoff, **(E)** SR, and **(F)** OSI. EDOF, extended depth of focus; MTF, modulation transfer function; OSI, objective scatter index; SR, Strehl ratio.

Regardless of the type of intraocular lens or axis of induced astigmatism, all the parameters of visual acuity and quality in our analysis declined with worsening astigmatism (Figure 6).

## Discussion

Residual astigmatism could affect retinal image quality by preventing light from focusing properly on the retina and resulting in blurred or distorted vision at all distances, and its impact on the retinal image quality of trifocal IOLs was the most pronounced when compared to EDOF and monofocal IOLs (12). Residual astigmatism affects a substantial proportion of patients after cataract surgery (4), prompting us to assess the tolerance of increasingly popular trifocal and EDOF lenses for astigmatism. Our analysis suggests that, regardless of the magnitude and direction of residual astigmatism, EDOF lenses are more tolerant to it. Both lens types are more tolerant to astigmatism when it is with or against the rule than when it is oblique. These results may help guide the choice of intraocular lens during cataract surgery.

We found that, depending on whether astigmatism was oblique, with the rule or against the rule, EDOF lenses tolerated astigmatism up to −1.00 DC or even −1.50 DC, whereas trifocal lenses tolerated astigmatism as strong as −1.00 DC only when astigmatism was with the rule. Our results are consistent with previous analyses suggesting that EDOF lenses can tolerate up to −1.50 DC (13), segmented refractive multifocal intraocular lenses, up to −1.00 DC (14); and diffractive multifocal lenses, up to −0.50 DC (9). One study has suggested that astigmatism worse than −1.00 DC should be corrected during implantation of multifocal intraocular lenses (15).

Not only the magnitude but also the direction of induced astigmatism affected visual acuity and quality after lens implantation in our sample, consistent with the vectorial nature of astigmatism (16), which must be considered during cataract surgery and corneal refractive surgery (17, 18). In fact, visual acuity in our subjects decreased with worsening severity of induced astigmatism, regardless of its axis. We also found that when the magnitude of astigmatism was held constant, its impact on visual acuity was smaller when its direction was with the rule than when it was oblique or against the rule, consistent with numerous studies in various countries about the effect of astigmatism on natural eyes (11, 19, 20), eyes that underwent laser refractive surgery (21), and pseudophakic eyes implanted with monofocal intraocular lenses (22, 23).

We found that induced astigmatism of −1.00 DC at an axis of 0° only slightly affected near, intermediate and distant vision, which is consistent with another study reporting that it reduced intermediate and distant vision less than at 90° (24). We did not find that astigmatism of −1.00 DC significantly improve near visual acuity due to the relatively small sample size, in contrast to a previous study (22, 25), although such astigmatism at an axis of 90° showed a tendency to improve near vision in some of our EDOF patients. The impact of astigmatism of −1.00 DC on near vision should be explored in larger samples. We found that such astigmatism reduced distant visual acuity, consistent with previous work (25).

Before induction of astigmatism, the trifocal group in our study showed abnormal values for all three visual quality parameters, whereas the EDOF group showed normal values. Nevertheless, the two groups did not differ significantly in uncorrected visual acuity or satisfaction with visual function. This may reflect that the diffractive trifocal lens creates a mismatch between objective visual quality outcomes and subjective visual perception. While this should be explored in future work, it may be explained, in part, by a previous finding that diffractive multifocal intraocular lenses display more stray light (26). The trifocal lens may generate more stray light because it has more rings than an EDOF lens, and the edge of each ring can give rise to stray light (27). Greater stray light may also explain why the other two visual quality parameters were worse in our trifocal group.

We found the impact of astigmatism at −1.50 and −2.00 DC to be substantial regardless of axis. We caution against interpreting this to mean that the impact is axis-independent, given the possibility that axial dependence was “drowned out” because of the strong magnitude. Future work should explore how the impact of mild astigmatism on objective visual quality depends on axis.

Our EDOF group reported lower incidence of glare, halos, and other optical phenomena than the trifocal group, which is consistent with previous studies (28, 29). Indeed, our trifocal group reported better near visual perception when performing fine tasks, based on the Visual Function-14 questionnaire. These findings may reflect that trifocal intraocular lenses are designed to provide distinct focal points for near, intermediate and distant vision. EDOF lenses, in contrast, are designed to offer moderate clarity at multiple distances, which may result in less sharp near vision.

Our comparison of the two types of intraocular lenses should be reliable because the two groups did not differ significantly in age, and none of the study participants had other eye comorbidities or history of ocular surgery. We set the artificial pupil diameter to 4 mm during all measurements, and we corrected refractive error before inducing astigmatism. In these ways, our two groups showed negligible differences in factors known to affect visual quality as measured using the Objective Quality Analysis System (30–32).

Our findings should be verified and extended in larger studies. Such work should also permit subgroup analysis to clarify, for example, potential relationships between astigmatism and types of adverse visual effects.

## Conclusion

Trifocal and EDOF intraocular lenses are less tolerant of oblique astigmatism than astigmatism with or against the rule. Both lens types are more tolerant of astigmatism with the rule than against it. EDOF lenses may provide better objective visual quality than trifocal lenses in the presence of astigmatism, regardless of its magnitude or axis. Although both intraocular lenses can give rise to glare and halos, they are associated with high satisfaction with vision and willingness among patients to recommend the same lens to others.

## Data Availability

The raw data supporting the conclusions of this article will be made available by the authors, without undue reservation.

## References

[1] Hashemi H Pakzad R Yekta A Aghamirsalim M Pakbin M Ramin S. Global and regional prevalence of age-related cataract: a comprehensive systematic review and meta-analysis. Eye. (2020) 34:1357–70. doi: 10.1038/s41433-020-0806-3, PMID: 32055021 PMC7376226

[2] Muñoz G Albarrán-Diego C Ferrer-Blasco T Sakla HF García-Lázaro S. Visual function after bilateral implantation of a new zonal refractive aspheric multifocal intraocular lens. J Cataract Refract Surg. (2011) 37:2043–52. doi: 10.1016/j.jcrs.2011.05.045, PMID: 22018366

[3] Alba-Bueno F Garzón N Vega F Poyales F Millán MS. Patient-perceived and laboratory-measured halos associated with diffractive bifocal and trifocal intraocular lenses. Curr Eye Res. (2018) 43:35–42. doi: 10.1080/02713683.2017.1379541, PMID: 29161162

[4] de Vries NE Webers CA Touwslager WR Bauer NJ de Brabander J Berendschot TT. Dissatisfaction after implantation of multifocal intraocular lenses. J Cataract Refract Surg. (2011) 37:859–65. doi: 10.1016/j.jcrs.2010.11.032 21397457

[5] Kim H Whang WJ Joo CK. Corneal astigmatism in patients after cataract surgery: a 10-year follow-up study. J Refract Surg. (2016) 32:404–9. doi: 10.3928/1081597X-20160303-01, PMID: 27304604

[6] Ang RE. Small-aperture intraocular lens tolerance to induced astigmatism. Clin Ophthal. (2018) 12:1659–64. doi: 10.2147/OPTH.S172557, PMID: 30233128 PMC6130293

[7] Ang RE. Comparison of tolerance to induced astigmatism in pseudophakic eyes implanted with small aperture, trifocal, or monofocal intraocular lenses. Clin Ophthal. (2019) 13:905–11. doi: 10.2147/OPTH.S208651, PMID: 31213762 PMC6549753

[8] Hayashi K Yoshida M Igarashi C Hirata A. Effect of refractive astigmatism on all-distance visual acuity in eyes with a trifocal intraocular Lens. Am J Ophthalmol. (2021) 221:279–86. doi: 10.1016/j.ajo.2020.07.051, PMID: 32777380

[9] Braga-Mele R Chang D Dewey S Foster G Henderson BA Hill W. Multifocal intraocular lenses: relative indications and contraindications for implantation. J Cataract Refract Surg. (2014) 40:313–22. doi: 10.1016/j.jcrs.2013.12.011, PMID: 24461503

[10] Khadka J Huang J Mollazadegan K Gao R Chen H Zhang S. Translation, cultural adaptation, and Rasch analysis of the visual function (VF-14) questionnaire. Invest Ophthalmol Vis Sci. (2014) 55:4413–20. doi: 10.1167/iovs.14-14017, PMID: 24917139

[11] Kobashi H Kamiya K Shimizu K Kawamorita T Uozato H. Effect of axis orientation on visual performance in astigmatic eyes. J Cataract Refract Surg. (2012) 38:1352–9. doi: 10.1016/j.jcrs.2012.03.032, PMID: 22727988

[12] Al-Amri SAJ Alió JL Milán-Castillo R D'Oria F Martinez-Abad A Yebana P. Clinical retinal image quality of a non-diffractive Wavefront-shaping extended depth of focus (Vivity) intraocular Lens. J Refract Surg. (2023) 39:103–10. doi: 10.3928/1081597X-20221130-04, PMID: 36779465

[13] Dick HB Piovella M Vukich J Vilupuru S Lin L. Prospective multicenter trial of a small-aperture intraocular lens in cataract surgery. J Cataract Refract Surg. (2017) 43:956–68. doi: 10.1016/j.jcrs.2017.04.038, PMID: 28823444

[14] Pedrotti E Bonacci E Alió Del Barrio JL Longo R Pagnacco C Marchini G. Astigmatism tolerance and visual outcomes after bilateral implantation of a hybrid continuous transitional focus IOL. J Refract Surg. (2023) 39:33–9. doi: 10.3928/1081597X-20221130-02, PMID: 36630438

[15] Hayashi K Manabe S Yoshida M Hayashi H. Effect of astigmatism on visual acuity in eyes with a diffractive multifocal intraocular lens. J Cataract Refract Surg. (2010) 36:1323–9. doi: 10.1016/j.jcrs.2010.02.016, PMID: 20656155

[16] Alpins NA. New method of targeting vectors to treat astigmatism. J Cataract Refract Surg. (1997) 23:65–75. doi: 10.1016/S0886-3350(97)80153-8, PMID: 9100110

[17] Reitblat O Gershoni A Mimouni M Vainer I Livny E Nahum Y. Refractive outcomes of high-magnitude astigmatism correction using femtosecond LASIK versus transepithelial PRK. Eur J Ophthalmol. (2021) 31:2923–31. doi: 10.1177/1120672120978885, PMID: 33295217

[18] Reitblat O Levy A Megiddo Barnir E Assia EI Kleinmann G. Toric IOL calculation in eyes with high posterior corneal astigmatism. J Refract Surg. (2020) 36:820–5. doi: 10.3928/1081597X-20200930-03, PMID: 33295994

[19] Casagrande M Baumeister M Bühren J Klaproth OK Titke C Kohnen T. Influence of additional astigmatism on distance-corrected near visual acuity and reading performance. Br J Ophthalmol. (2014) 98:24–9. doi: 10.1136/bjophthalmol-2013-303066, PMID: 23703094

[20] Remón L Tornel M Furlan WD. Visual acuity in simple myopic astigmatism: influence of cylinder axis. Optom Vis Sci. (2006) 83:311–5. doi: 10.1097/01.opx.0000216099.29968.36, PMID: 16699444

[21] Mimouni M Nemet A Pokroy R Sela T Munzer G Kaiserman I. The effect of astigmatism axis on visual acuity. Eur J Ophthalmol. (2017) 27:308–11. doi: 10.5301/ejo.5000890 27739560

[22] Trindade F Oliveira A Frasson M. Benefit of against-the-rule astigmatism to uncorrected near acuity. J Cataract Refract Surg. (1997) 23:82–5. doi: 10.1016/S0886-3350(97)80155-1 9100112

[23] Serra P Chisholm C Sanchez Trancon A Cox M. Distance and near visual performance in pseudophakic eyes with simulated spherical and astigmatic blur. Clin Exp Optom. (2016) 99:127–34. doi: 10.1111/cxo.12350, PMID: 26840890

[24] Rementería-Capelo LA Contreras I García-Pérez JL Blázquez V Ruiz-Alcocer J. Effect of residual astigmatism and defocus in eyes with trifocal intraocular lenses. J Cataract Refract Surg. (2022) 48:679–84. doi: 10.1097/j.jcrs.0000000000000814, PMID: 34508029

[25] Singh A Pesala V Garg P Bharadwaj SR. Relation between uncorrected astigmatism and visual acuity in pseudophakia. Optom Vis Sci. (2013) 90:378–84. doi: 10.1097/OPX.0b013e318288afb5, PMID: 23458979

[26] Ehmer A Rabsilber TM Mannsfeld A Sanchez MJ Holzer MP Auffarth GU. Influence of different multifocal intraocular lens concepts on retinal stray light parameters. Ophthalmologe. (2011) 108:952–6. doi: 10.1007/s00347-011-2411-0, PMID: 21853217

[27] Hecht I Kanclerz P Tuuminen R. Secondary outcomes of lens and cataract surgery: more than just “best-corrected visual acuity”. Prog Retin Eye Res. (2023) 95:101150. doi: 10.1016/j.preteyeres.2022.101150, PMID: 36481168

[28] Böhm M Hemkeppler E Kohnen T. Self-rated quality of vision and optical phenomena intensity of diffractive presbyopia-correcting IOLs: EDoF, trifocal vs. panfocal. J Cataract Refract Surg. (2022) 48:877–86. doi: 10.1097/j.jcrs.0000000000000862, PMID: 34753879

[29] Farvardin M Johari M Attarzade A Rahat F Farvardin R Farvardin Z. Comparison between bilateral implantation of a trifocal intraocular lens (Alcon Acrysof IQ^®^ PanOptix) and extended depth of focus lens (Tecnis^®^ Symfony^®^ ZXR00 lens). Int Ophthalmol. (2021) 41:567–73. doi: 10.1007/s10792-020-01608-w, PMID: 33040273

[30] Carkeet A Leo SW Khoo BK Au Eong KG. Modulation transfer functions in children: pupil size dependence and meridional anisotropy. Invest Ophthalmol Vis Sci. (2003) 44:3248–56. doi: 10.1167/iovs.02-1064, PMID: 12824277

[31] Miao H Tian M He L Zhao J Mo X Zhou X. Objective optical quality and intraocular scattering in myopic adults. Invest Ophthalmol Vis Sci. (2014) 55:5582–7. doi: 10.1167/iovs.14-14362, PMID: 25103263

[32] Zhang XF Qiao LY Cai XG Li XX Tan JX Guan Z. Analysis of related factors of optical quality in healthy Chinese adults: a community-based population study. Chin Med J. (2020) 133:2308–14. doi: 10.1097/CM9.0000000000000994, PMID: 32868501 PMC7546838

